# Chloroplast SRP54s are Essential for Chloroplast Development in Rice

**DOI:** 10.1186/s12284-020-00415-2

**Published:** 2020-08-06

**Authors:** Yongfeng Shi, Yan He, Xiangguang Lv, Yanlin Wei, Xiaobo Zhang, Xia Xu, Liangjian Li, Jian-li Wu

**Affiliations:** 1 State Key Laboratory of Rice Biology, China National Rice Research Institute, Hangzhou, 310006 China; 2 National Key Facility for Crop Gene Resources and Genetic Improvement, Institute of Crop Sciences, Chinese Academy of Agricultural Sciences, Beijing, 100081 China

**Keywords:** Rice, Pale green leaf, Chloroplast, Signal recognition particle, Map-based cloning

## Abstract

**Background:**

The chloroplast signal recognition particle 54 (cpSRP54) is known for targeting the light-harvesting complex proteins to thylakoids and plays a critical role for chloroplast development in Arabidopsis, but little is known in rice. Here, we reported two homologous cpSRP54s that affect chloroplast development and plant survival in rice.

**Results:**

Two rice *cpSRP54* homologues, *OscpSRP54a* and *OscpSRP54b*, were identified in present study. The defective *OscpSRP54a* (*LOC_Os11g05552*) was responsible for the pale green leaf phenotype of the viable *pale green leaf 14* (*pgl14*) mutant. A single nucleotide substitution from G to A at the position 278, the first intron splicing site, was detected in *LOC_Os11g05552* in *pgl14*. The wild type allele could rescue the mutant phenotype. Knockout lines of *OscpSRP54b* (*LOC_Os11g05556*) exhibited similar pale green phenotype to *pgl14* with reduced chlorophyll contents and impaired chloroplast development, but showed apparently arrested-growth and died within 3 weeks. Both *OscpSRP54a* and *OscpSRP54b* were constitutively expressed mainly in shoots and leaves at the vegetative growth stage. Subcellular location indicated that both OscpSRP54a and OscpSRP54b were chloroplast-localized. Both OscpSRP54a and OscpSRP54b were able to interact with OscpSRP43, respectively. The transcript level of *OscpSRP43* was significantly reduced while the transcript level of *OscpSRP54b* was apparently increased in *pgl14.* In contrast, the transcript levels of *OscpSRP54a*, *OscpSRP43* and *OscpSRP54b* were all significantly decreased in *OscpSRP54b* knockout lines.

**Conclusion:**

Our study demonstrated that both OscpSRP54a and OscpSRP54b were essential for normal chloroplast development by interacting with OscpSRP43 in rice. OscpSRP54a and OscpSRP54b might play distinct roles in transporting different chloroplast proteins into thylakoids through cpSRP-mediated pathway.

## Background

Chloroplasts are the site for photosynthesis and other important metabolic processes such as fatty acid and amino acid biosynthesis (Nelson and Ben-Shem 2004; Lopez-Juez and Pyke 2005). The organelle contains up to several thousands of proteins, the majority of which are encoded in the nucleus with only a small fraction encoded in the plastid genome both in Arabidopsis and rice (Abdallah et al. 2000; Richly and Leister 2004). The nucleus-encoded chloroplast proteins are thus required to be transferred into the chloroplasts usually depending on the Toc/Tic complexes on the inner and outer chloroplast membranes (Jarvis and Robinson 2004; Schwenkert et al. 2011). Once these proteins enter into the chloroplast stroma, they may take on the final form, or further transport into the thylakoids through four distinct transport pathways namely, cpSec, ΔpH/Tat, cpSRP and spontaneous pathways (Schünemann 2007; Jarvis 2008).

The chloroplast signal recognition particle (cpSRP) and its receptor (cpFtsY) are involved in transporting of chloroplast proteins such as mature light-harvesting chlorophyll a/b binding proteins (LHCPs) to thylakoid membranes (Akopian et al. 2013). Defective or deficient in cpSRP and cpFtsY would impair the biogenesis of chloroplasts, leading to chlorophyll degeneration and chlorotic leaves in plants (Amin et al. 1999; Klimyuk et al. 1999; Asakura et al. 2004; Rutschow et al. 2008; Lv et al. 2015). In Arabidopsis, two null mutants of *cpSRP54*, *ffc1–1* and *ffc1–2*, produce yellow first true leaves that become green subsequently. The levels of reaction center proteins are significantly lower in the young *ffc1–2* leaves but recover to the normal protein level in adult plants grown on agar plates (Amin et al. 1999). However, the *ffc1–2* plants are pale green with a fewer number of leaves and reduced rosette diameter grown under soil conditions (Rutschow et al. 2008). The null mutant of Arabidopsis *cpSRP43*, *chaos*, exhibits pale green leaves including the first true leaves in the whole lifecycle, with elevated chlorophyll a/b ratio, but a normal level of reaction center proteins (Amin et al. 1999; Klimyuk et al. 1999). The double mutant *ffc/chaos* exhibits pale yellow leaves at all growth stages with drastically reduced levels of LHCPs except Lhcb4 (Hutin et al. 2002). Similarly, the chlorophyll synthesis and chloroplast development are impaired in company with altered expression of chlorophyll synthesis-associated genes in rice *OscpSRP43* mutants, w67 and *pgl3*, both of them show pale green leaves at all growth stages (Lv et al. 2015; Ye et al. 2018). Furthermore, high temperatures inhibit plant growth and facilitate the progression of leaf senescence in *pgl3* (Ye et al. 2018). The maize *cpFtsY* mutant *csr1–1* exhibits a pale yellow-green phenotype while *csr1–3* shows a slight pale green phenotype (Asakura et al. 2004). Interestingly, both *csr1–1* and *csr1–2* are seedling lethal, similar to the Arabidopsis mutants *cpFtsY-1* and *cpFtsY-2,* with completely arrested-growth at the cotyledon or the first true leaf stage under photoautotrophic conditions (Asakura et al. 2004; Asakura et al. 2008). It has been shown that the mutation lines of cpSRP pathway genes are able to accumulate truncated light-harvesting chlorophyll antenna (TLA) and enhance energy conversion efficiency in high-density cultures under bright sunlight conditions in *Chlamydomonas reinhardtii* (Kirst and Melis 2014; Jeong et al. 2017). Similar phenomena have been observed in tobacco, the *cpSRP43* knockdown plants show lower chlorophyll contents in the RNAi canopy leaves with increased leaf-to-stem ratio, improved photosynthetic productivity and canopy biomass accumulation under high-density cultivation conditions (Kirst et al. 2018).

We previously identified a stable-inherited rice *pale green leaf 14* (*pgl14*) mutant (originally termed HM14) at the vegetative stage (Shi et al. 2013). In this study, we isolated *PGL14* that encoded for cpSRP54 (hereafter cpSRP54a). A single base substitution in the mutant allele resulting in an altered mRNA splicing is responsible for the pale green phenotype confirmed by genetic complementation. We also isolated a homologue of *PGL14*, *LOC_Os11g05556* (hereafter cpSRP54b). Although the knockout lines of *cpSRP54b* displayed a similar pale green leaf phenotype to *pgl14* with reduced chlorophyll levels, impaired chloroplast structures and down-regulated expression of chlorophyll synthesis/development related genes, they were seedling lethal. Both cpSRP54a and cpSRP54b were chloroplast-localized and could interact with OscpSRP43, respectively. Our results indicated that both of them are required for chloroplast development by interacting with cpSRP43 to potentially participate in protein transport into thylakoids in rice.

## Results

### Map-Based Isolation of *PGL14*

**Fig. 1:**
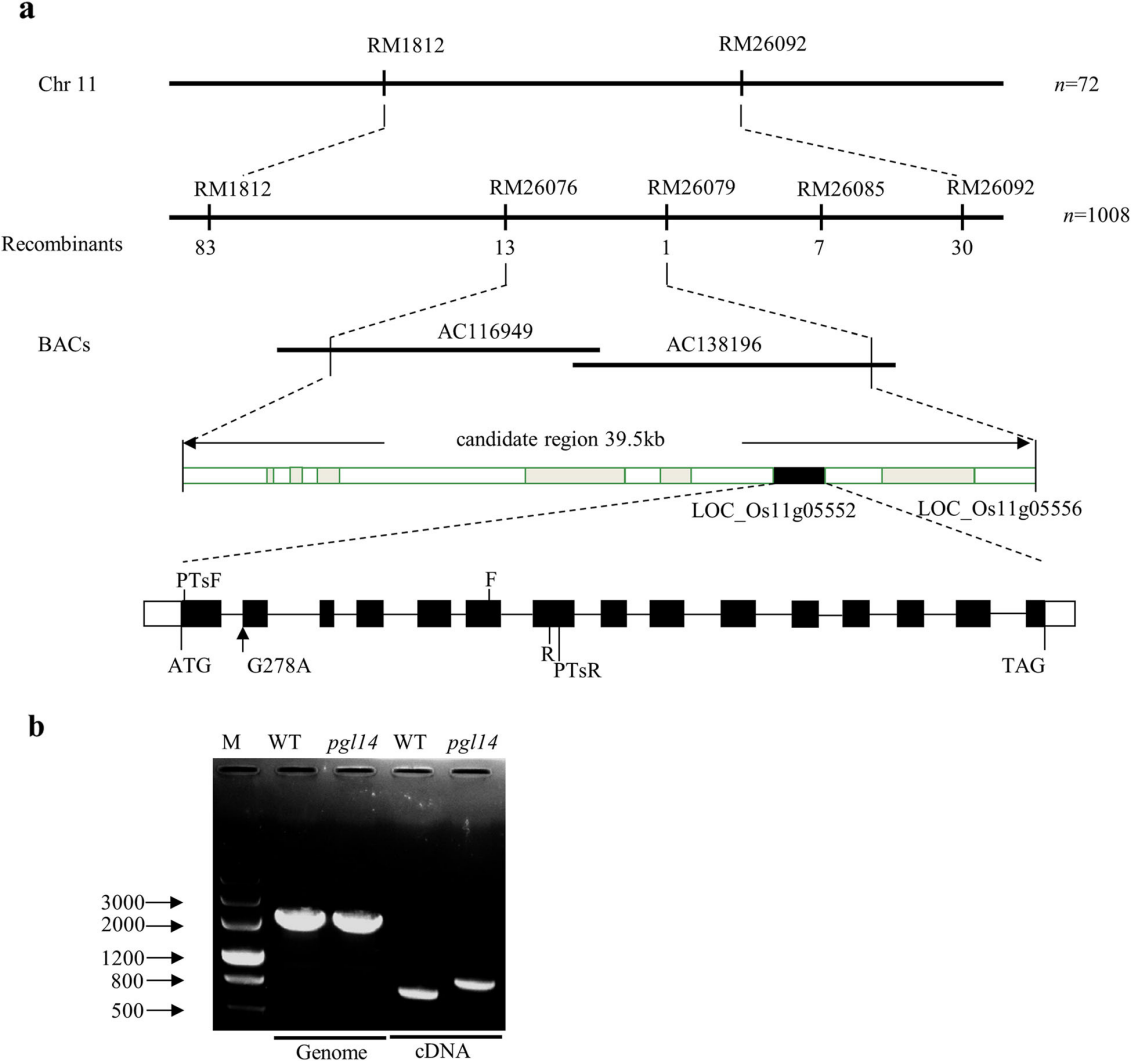
Map-based cloning of *PGL14.***a***PGL14* localizes to the short arm on chromosome 11 between RM26076 and RM26079, and is narrowed down to a 39.5 kb region covering the bacterial artificial chromosome clones AC116949 and AC138196. The 39.5 kb region contains 7 putative ORFs, the black box indicates *LOC_Os11g05552*, gray boxes indicate the other ORFs. *LOC_Os11g05552* consists of 15 exons and 14 introns indicated as blank boxes and lines, respectively. The black arrow indicates the point mutation (G278A) at 1st intron splicing site. PTsF and PTsR are the forward and reverse primers for PCR analysis in (**b**). F and R are forward and reverse primers for qRT-PCR analysis; **b** RT-PCR shows different transcripts from WT and *pgl14*. The genomic DNA was used as a control

**Fig. 2:**
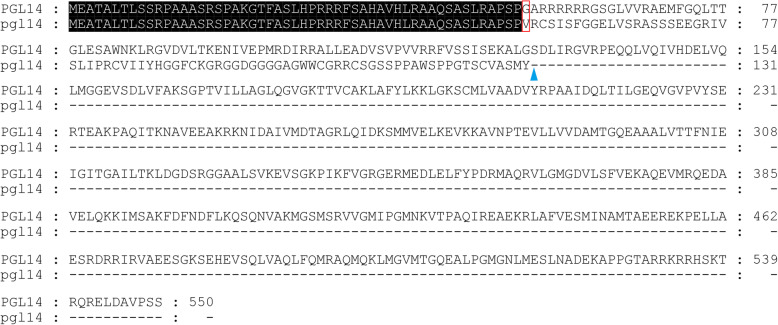
Alignment of the amino acid sequences of *LOC_Os11g05552* in WT and *pgl14.* pgl14 is the predicted amino acid sequence deduced from abnormal transcript detected in *pgl14.* Frame shift in pgl14 is indicated by the red box. Blue arrow indicates the premature termination in pgl14

We previously identified a chlorophyll-deficient mutant *pgl14* exhibiting pale green leaf phenotype from the first leaf to the flag leaf under natural conditions and mapped the recessive mutation to a 299 kb region in chromosome 11 (Shi et al. 2013). To fine map the mutation, a total of 1008 mutant-type F_2_ individuals derived from the cross *pgl14*/Moroberekan were used for genotyping. The *PGL14* locus was further narrowed down to a 39.5 kb genomic region between RM26076 and RM26079, covering the BAC clones AC116949 and AC138169 (Fig. 1a). Seven open reading frames (ORFs) were annotated within this region in the database of Rice Genome annotation Project (http://rice.plantbiology.msu.edu/cgi-bin/gbrowse/rice/), two of which (*LOC_Os11g05552* and *LOC_Os11g05556*) were both annotated as hypothetical loci encoding for cpSRP54. Sequence analysis showed that a single nucleotide substitution from G to A at position 278 was detected in *LOC_Os11g05552* in *pgl14*, and the mutation localized to the predicted splicing site on the last nucleotide of the first intron. RT-PCR analysis showed that the transcript in *pgl14* was longer than that of WT, confirming the presence of the altered splicing transcript in the mutant (Fig. 1b). Sequence analysis also showed that the first intron of 119 bp was maintained in the mutant transcript which had a frame shift starting from valine at position 54 and terminating prematurely at position 131 (Fig. 2, Genbank accession MN105082). Therefore, *LOC_Os11g05552* was most likely the candidate gene of *PGL14*.

### *OscpSRP54a* Rescues the Pale Green Phenotype

**Fig. 3:**
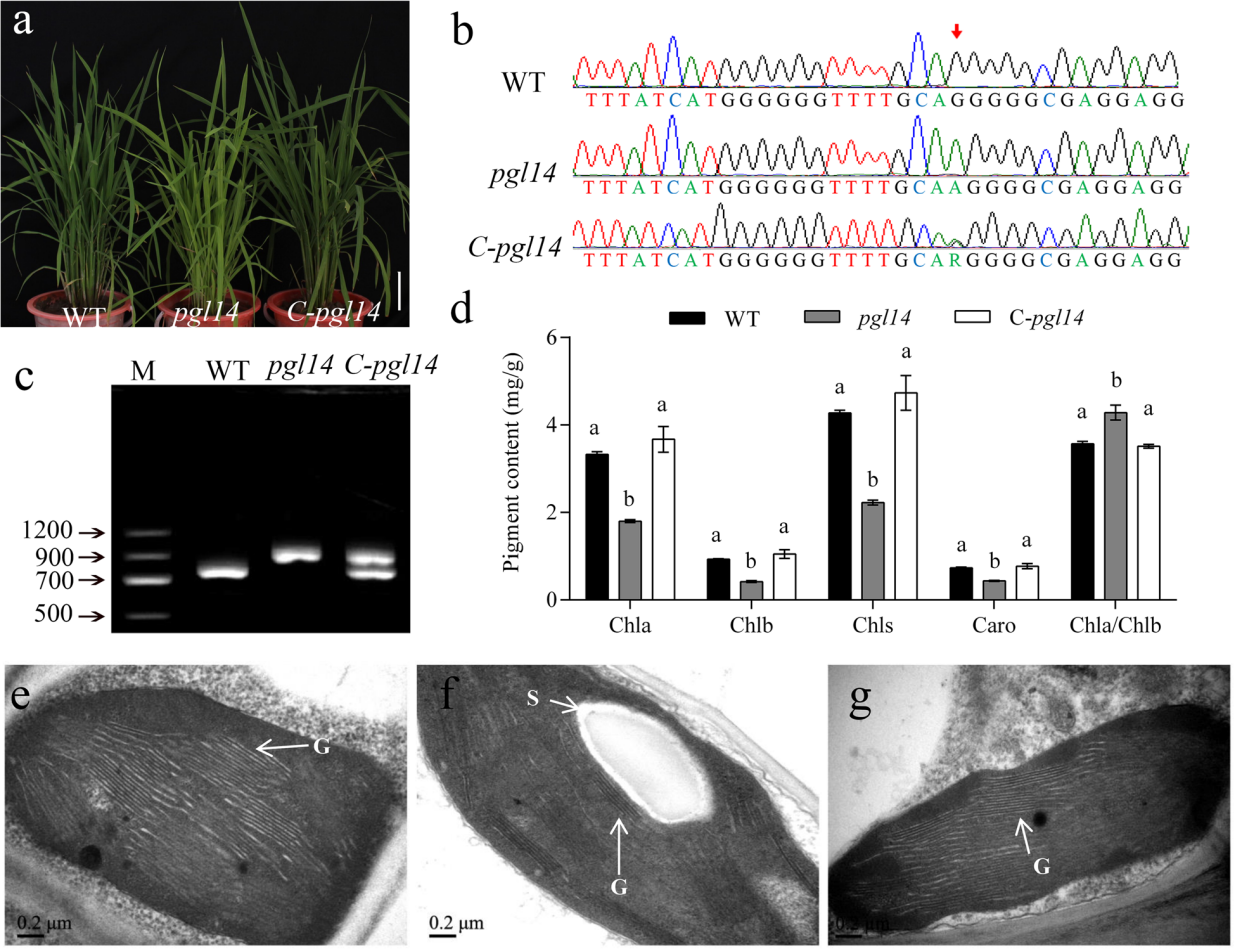
Functional complementation of *OscpSRP54a*. **a** Phenotype of WT, *pgl14* and complementary line C-*pgl14*. Bar = 20 cm; **b** Sequence analysis of the mutation site (red arrow) in WT, *pgl14* and C-*pgl*14; **c***OscpSRP54a* transcripts in WT, *pgl14* and C-*pgl14*; **d** Pigment contents in WT, *pgl14* and C-*pgl14* in 8-week-old leaves. Data are means ± SD (*n* = 3). Means with different letters indicate significant differences according to One-way ANOVA and Duncan’s test (*p* ≤ 0.01). Chloroplast ultrastructure of WT (**e**), *pgl14* (**f**) and C-*pgl14* (**g**) at the tillering stage. G, grana thylakoid; S, starch granule

To verify the function of *PGL14*, the complementation vector cPGL carrying the entire coding region of *PGL14*, a 3.6 kb of upstream sequence and a 1.5 kb of downstream sequence was transformed into the rice calli induced from *pgl14* mature embryo through *Agrobacterium tumefaciens*-mediated transformation. Five independent transgenic lines were obtained and showed the normal green phenotype similar to WT (Fig. 3a). Sequence characterization of these transgenic plants indicated that the complementary plants displayed a double peak (A and G) while *pgl14* and WT presented a single peak, respectively (Fig. 3b), indicating that the wild-type allele has been incorporated into the mutant genome. Furthermore, RT-PCR analysis confirmed that both transcripts were presented in the transgenic plants (Fig. 3c). For the pigment levels, *pgl14* showed apparently lowered Chl a, Chl b, carotenoid (Caro) and total chlorophyll (Chls) contents with significantly elevated Chl a/b ratio compared with WT, and all these parameters recovered to WT levels in complementary lines (Fig. 3d).. In addition, the chloroplast ultrastructure of complementary line C-*pgl14* was similar to that of WT, displaying normal thylakoid membranes and stacked grana (Fig. 3e-g). Taken together, *PGL14* was indeed the target gene responsible for the pale green leaf phenotype in *pgl14*, hereafter *PGL14* is termed *OscpSRP54a.*

### cpSRP54s are Conserved in Plants

**Fig. 4:**
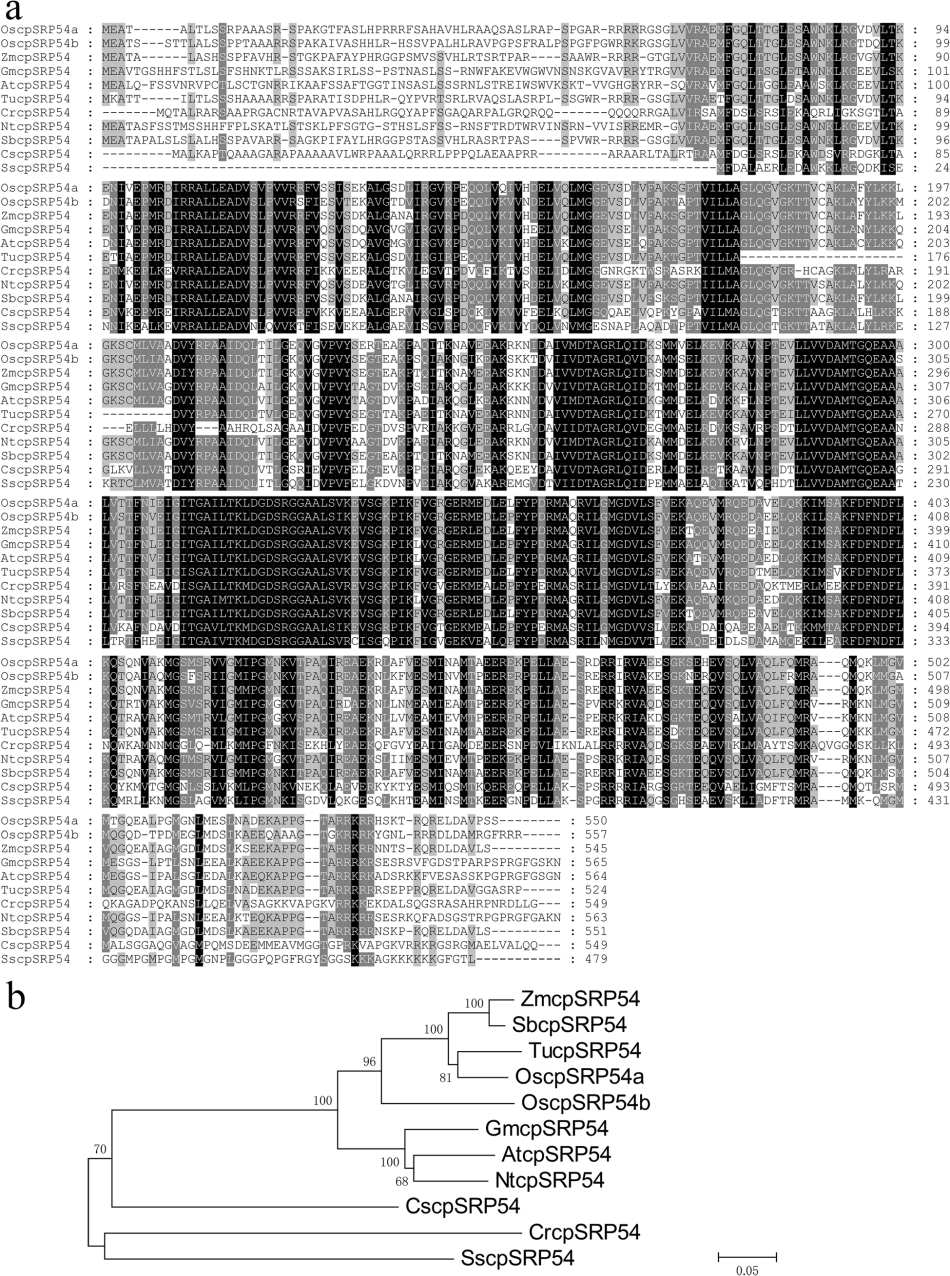
Sequence comparison and phylogenetic tree of OscpSRP54a homologues. **a** Amino acid sequence comparison of OscpSRP54a homologues. Amino acid residues that are identical or similar are shaded in black and gray, respectively; **b** Phylogenetic tree of OscpSRP54a homologues including ZmcpSRP54 (*Zea mays*, XP_008679417), TucpSRP54 (*Triticum urartu*, EMS61888), GmcpSRP54 (*Glycine max*, XP_003521470), AtcpSRP54 (*Arabidopsis thaliana*, AAC64139), CrcpSRP54 (*Chlamydomonas reinhardtii*, AAK12834), NtcpSRP54 (*Nicotiana tabacum*, XP_016448785), SbcpSRP54 (*Sorghum bicolor*, KXG27759), OscpSRP54b (*Oryza sativa* L, ABG22368), CscpSRP54 (*Chlorella sorokiniana*, PRW50902), SscpSRP54 (*Spirulina subsalsa*, WP_017306040)

The cpSRP54 is widely present in photoautotrophic organisms. To simplify the phylogenetic analysis, we chose 11 OscpSRP54a homologues representing 10 species including the monocot, dicot and algae. Blastp analysis showed that OscpSRP54a shared 47–86% identity at the amino acid level with various cpSRP54 from *Chlamydomonas reinhardtii* (47%), *Arabidopsis thaliana* (70%), *Glycine max* (75%), *Nicotiana tobacum* (79%), *Triticum urartu* (81%), *Zea mays* (85%), *Sorghum bicolor* (86%), *Chlorella sorokiniana* (57%) and *Spirulina subsalsa* (55%) respectively (Fig. 4a). In addition, OscpSRP54a has 78% amino acid identity to OscpSRP54b which possess 47–78% amino acid identity with cpSRP54 from the other species. Apparently, the more closer relationship, the higher the amino acid identity shows in various cpSRP54s in which the function required for chloroplast development has been extensively elaborated in *Arabidopsis* and *C. reinhardtii* (Li et al. 1995; Yu et al. 2012; Jeong et al. 2017). The results indicated that cpSRPs were highly conserved in plant species.

To reveal the evolutionary relationship of the cpSRP54 homologues, a phylogenetic tree was constructed. The results showed that cpSRP54 homologues in the higher plants could be classified into two groups as the monocot and dicot (Fig. 4b). Both OscpSRP54a and OscpSRP54b are clustered in the monocot group, however, OscpSRP54a is more closely related to TucpSRP54, ZmcpSRP54 and SbcpSRP54 than OscpSRP54b, probably indicating that OscpSRP54a and OscpSRP54b were likely originated differently.

### *OscpSRP54b* is Essential for Chloroplast Development

**Fig. 5:**
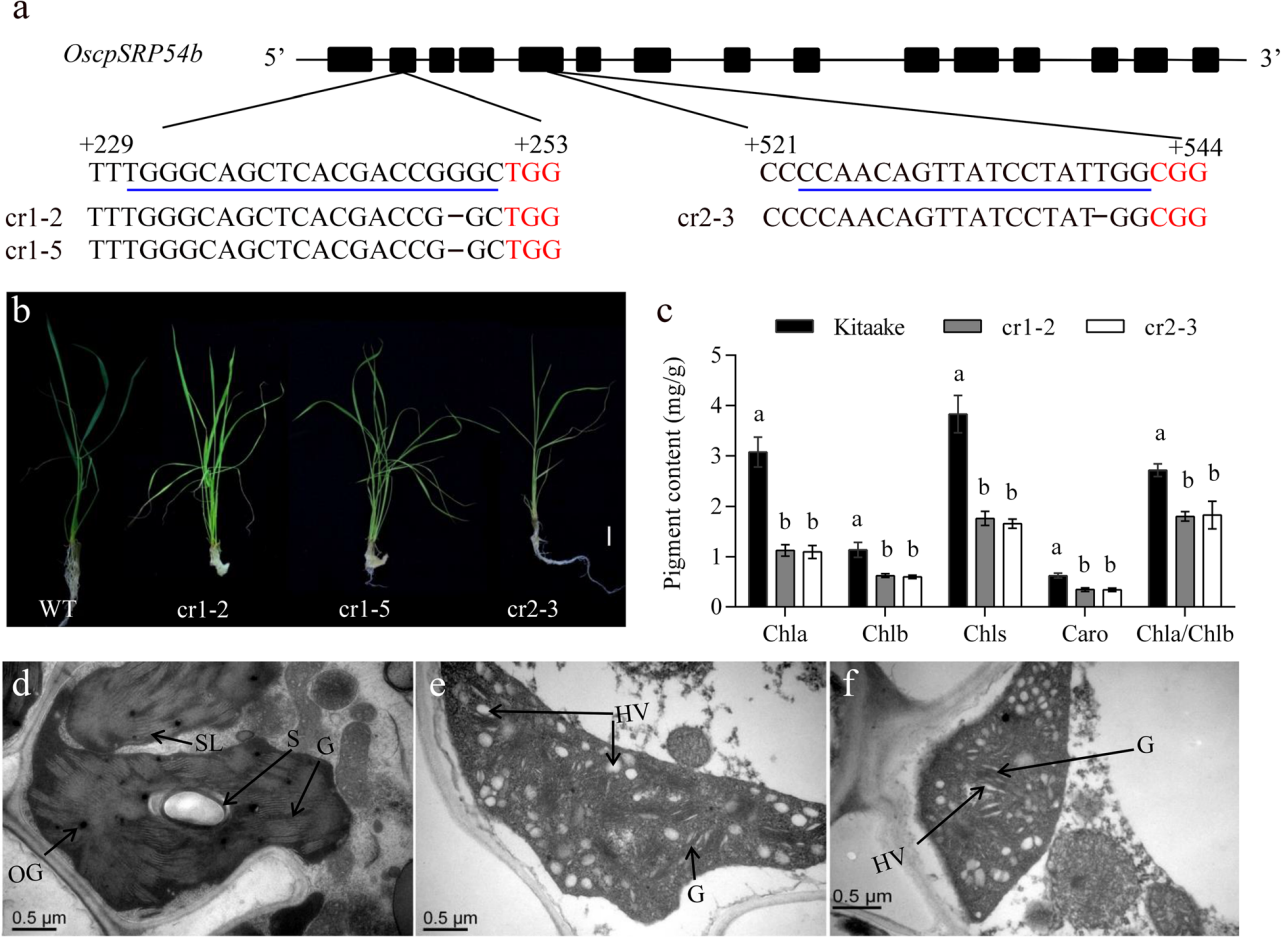
Functional verification of *OscpSRP54b* for chloroplast development. **a** Deletion mutation at the target site in three representative knockout lines generated by the CRISPR/Cas9-mediated editing. cr1–2, cr1–5 and cr2–3 are homozygous mutants carrying 1-bp deletion on both homochromosomes. Black boxes indicate exons and lines indicate introns of *OscpSRP54b*. The sgRNA target sequence is underlined in blue and the PAM motif is highlighted in red letters, F and R are the forward and reverse primers for qRT-PCR analysis; **b** Phenotype of Kitaake and *OscpSRP54b* knockout mutants. Bar = 2 cm; **c** Pigment contents in 1 week-old leaves of Kitaake, cr1–2 and cr2–3. Different letters indicate significant differences according to One-way ANOVA and Duncan’s test (*p* ≤ 0.01); Chloroplast ultrastructure of Kitaake (**d**), cr1–2 (**e**) and cr2–3 (**f**). G, grana thylakoid; S, starch granule; OG, osmiophilic plastoglobuli; SL, stroma lamellae; HV, hollow vesicle

To verify whether *OscpSRP54b* functions similar to *OscpSRP54a*, we introduced the CRISPR/Cas9 vectors cr1 and cr2 into Kitaake-derived embryogenic calli to edit the two specific target sites in exon 2 and exon 5, respectively (Fig. 5a). A total of 7 and 5 independent T_0_ transgenic lines were obtained from construct cr1 and cr2, respectively. Two (cr1–2 and cr1–5) out 7 T_0_ lines from construct cr1 were homozygous and exhibited pale green phenotype similar to *pgl14* (Fig. 5b). Sequence analysis confirmed that there was one base deletion at the target site in cr1–2 and cr1–5, respectively (Fig. 5a). One (cr2–3) out of 5 T_0_ lines from construct cr2 was homozygous and showed pale green leaf phenotype similar to *pgl14* (Fig. 5b), and sequence analysis showed that cr2–3 had a single nucleotide deletion at the target site (Fig. 5a). We then further characterized the performance of cr1–2 and cr2–3. Firstly, we determined the levels of photosynthetic pigments, and found that the contents of Chl a, Chl b, total Chls and carotenoid in cr1–2 and cr2–3 were significantly lower than those of Kitaake, and the Chl a/b ratio of both knockout mutants were much lower than in the WT (Fig. 5c). Then we observed the chloroplast ultrastructure by transmission electron microscopic analysis. The results showed that both cr1–2 and cr2–3 possessed a large number of hollow vesicles, reduced number of grana, irregular grana thylakoids, and destroyed stromal lamella compared with Kitaake (Fig. 5d-f). Moreover, cr1–2, cr2–3 as well as cr1–5 showed apparently arrested-growth and died within 3 weeks at the seedling stage. These results suggested that *OscpSRP54b* was essential for the chloroplast development and plant survival in rice.

### Rice *cpSRPs* are Mainly Expressed in Shoots and Leaves and cpSRPs Localize to Chloroplasts

**Fig. 6:**
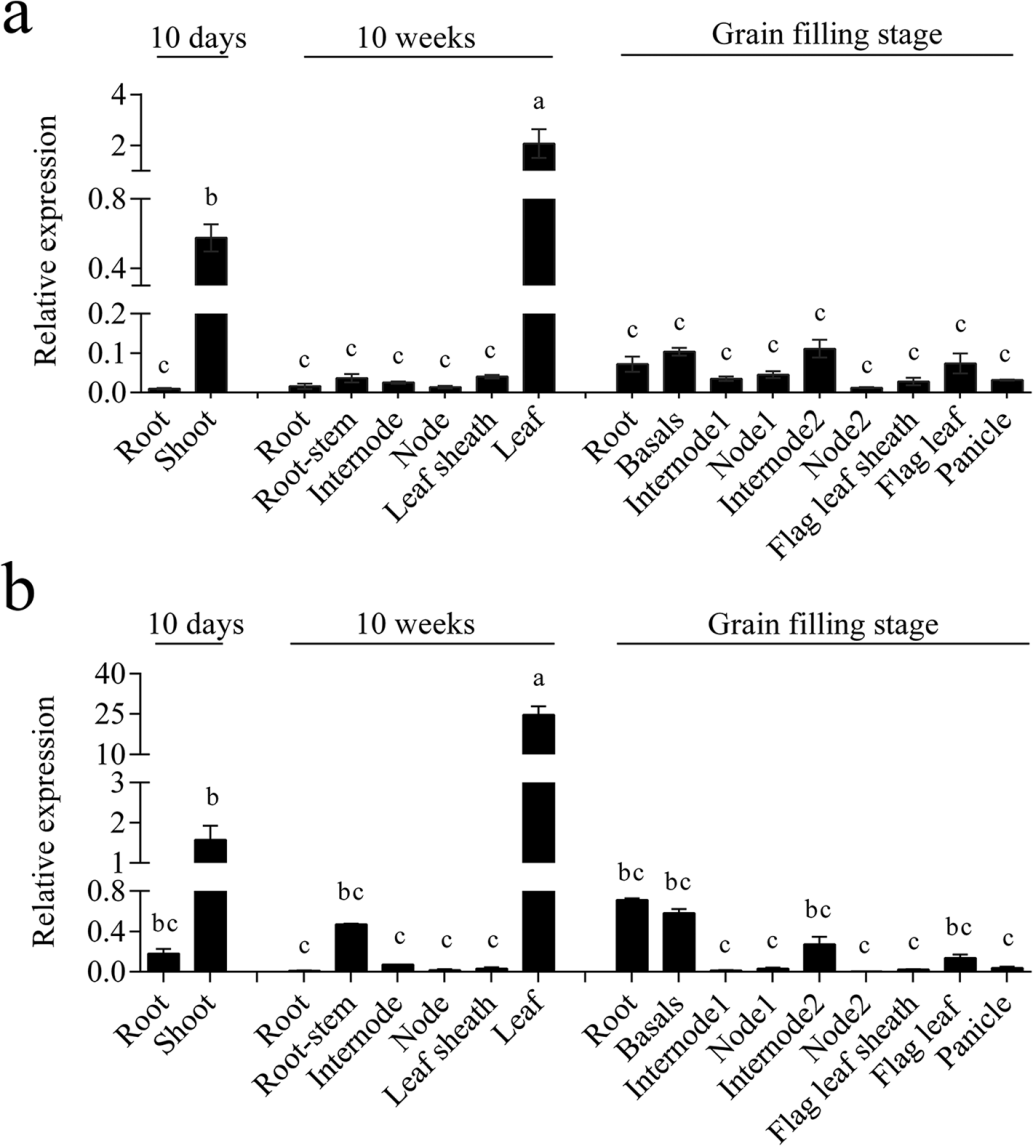
Spatial and temporal expression of *OscpSRP54a* and *OscpSRP54b*. **a** Relative expression levels of *OscpSRP54a* in various tissues of IR64 at different growth stages; **b** Relative expression levels of *OscpSRP54b* in various tissues of Kitaake at different growth stages. Different letters indicate significant differences according to One-way ANOVA and Duncan’s test (*p* ≤ 0.01)

To examine the expression pattern of *OscpSRP54a* and *OscpSRP54b*, qRT-PCR was carried out using samples from various tissues at the germination, tillering and heading stages. The results showed that the expressions of *OscpSRP54a* and *OscpSRP54b* were detected in all tissues tested with the highest expression levels of *OscpSRP54a* and *OscpSRP54b* in the leaves at the tillering stage, followed by the shoots at the germination stage (Fig. 6). Our results indicated that the expression patterns were similar between the two genes which were mainly expressed in above ground parts of the plants.

**Fig. 7:**
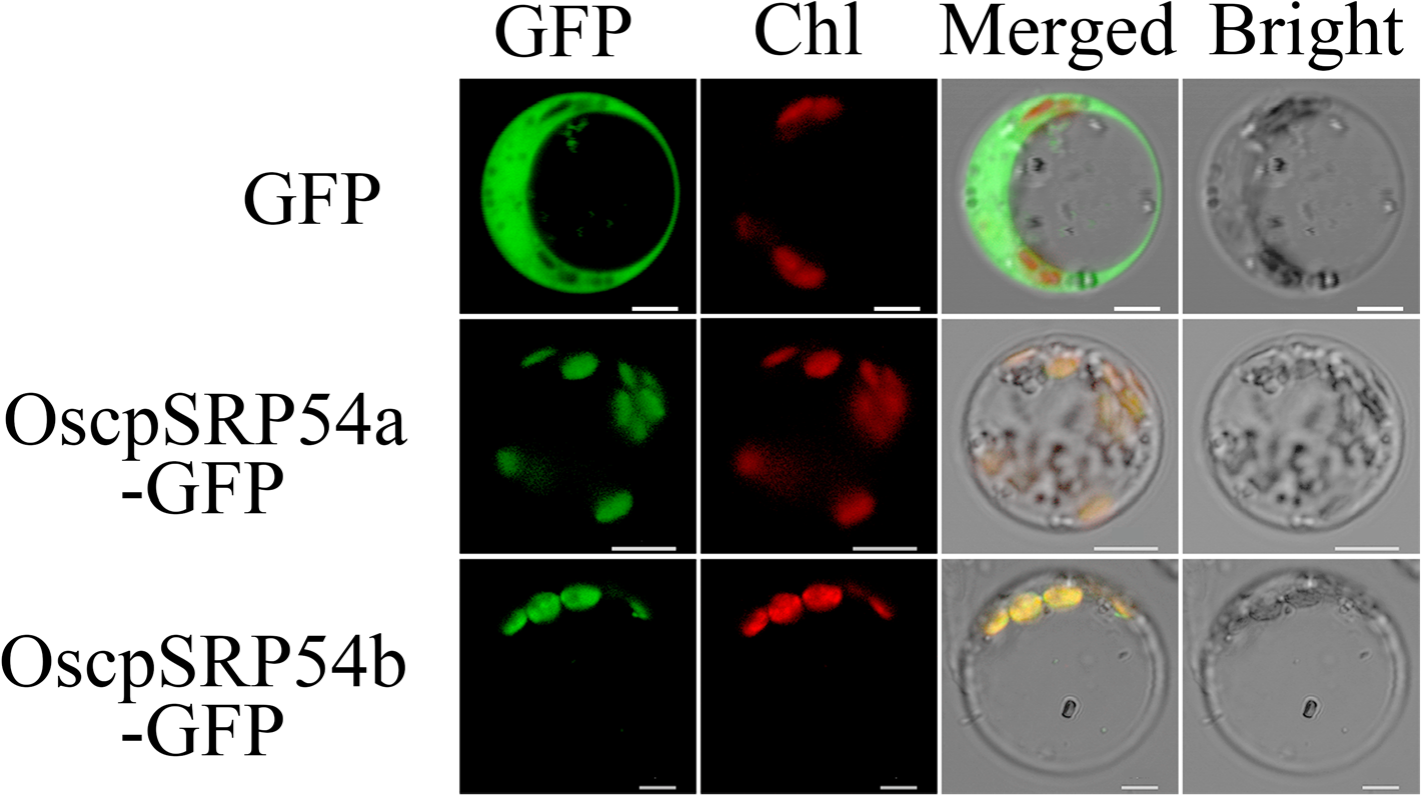
Subcellular localization of OscpSRP54a and OscpSRP54b in rice protoplasts. Green fluorescence indicates GFP signal, red fluorescence indicates chloroplast autofluorescence, and yellow fluorescence indicates the merged signal of green and red fluorescence

To determine the subcellular location, we first predicted their physical locations using the ChloroP program (http://www.cbs.dtu.dk/services/ChloroP) and the results showed that both OscpSRP54a and OscpSRP54b were located in chloroplasts (Supplementary Fig. 1). The subcellular localization of OscpSRP54a and OscpSRP54b were then confirmed by expressing the constructs PAN580-OscpSRP54a and PAN580-OscpSRP54b in protoplasts. The green fluorescence signals of OscpSRP54a:GFP fusion protein and OscpSRP54b:GFP fusion protein overlapped with the chlorophyll autofluorescence signal whereas the free GFP signal was observed in the cytoplasm and nucleus (Fig. 7). These results demonstrated that both OscpSRP54a and OscpSRP54b were chloroplast-targeted proteins.

### Down-Regulated Expression of *OscpSRP43* in *pgl14* and cr1–2

**Fig. 8:**
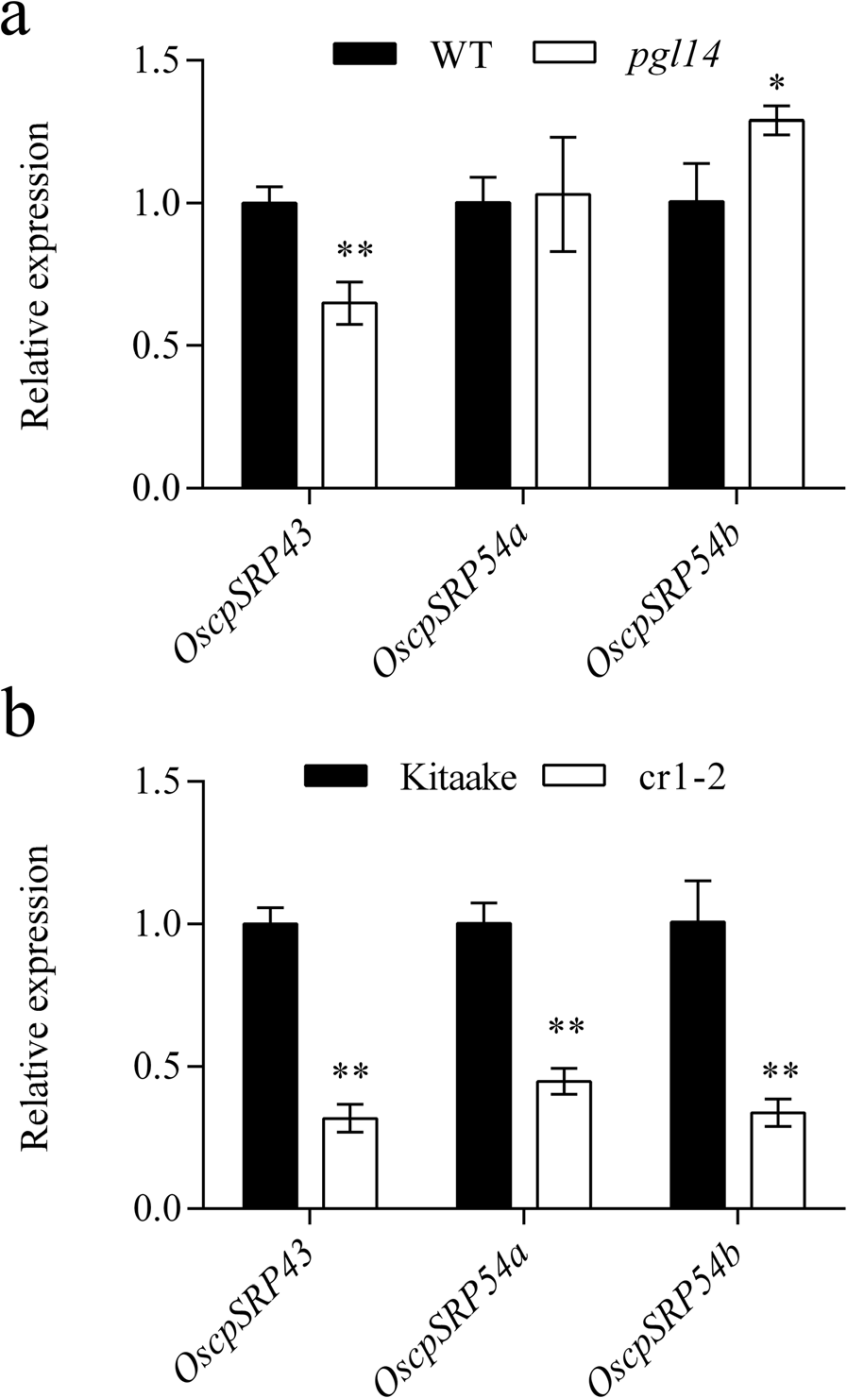
Differential expression of cpSRP genes. **a** Transcript levels of cpSRP genes in the wild type IR64 and *pgl14* at 3 weeks after sowing. **b** Transcript levels of cpSRP genes in the wild type Kitaake and cr1–2 at 1 week after transplanting. The transcript levels of tested genes are normalized to the levels of *Ubq* (*LOC_Os03g13170*). The values are means ± SD (*n* = 3). The asterisk indicates the significant difference between the wild type and mutant according to Student’s *t*-test (* *p* ≤ 0.05; ** *p* ≤ 0.01)

It has been shown that cpSRP43 interacts with cpSRP54 and is critical for chloroplast development by transporting proteins to thylakoids (Schünemann 2007; Akopian et al. 2013). To investigate whether the expression of *cpSRP43* was affected in *pgl14* and cr1–2, we measured the transcript levels of *OscpSRP43*, *OscpSRP54a* and *OscpSRP54b* by qRT-PCR. The results showed that the transcript level of *OscpSRP43* was significantly decreased in *pgl14* compared with WT, in contrast, the expression level of *OscpSRP54b* was significantly increased in *pgl14* compared with WT, whereas the expression level of *OscpSRP54a* was similar between *pgl14* and WT (Fig. 8a). In addition, the transcript levels of *OscpSRP43*, *OscpSRP54a* and *OscpSRP54b* were all notably reduced in cr1–2 compared with Kitaake (Fig. 8b). The results suggested that both mutations of *cpSRP54a* and *cpSRP54b* resulted in down-regulated expression of *cpSRP43* in rice.

The expression of genes associated with chlorophyll biosynthesis and chloroplast development was examined in *pgl14* and cr1–2. The results showed that expression profile of most genes (such as *PsbA*, *YGL1* and *ChlD*) was altered between the mutant and its wild type (Supplementary Fig. 2). These results further indicated that both *OscpSRP54a* and *OscpSRP54b* played an important role for normal development of chloroplasts in rice.

### Both *OscpSRP54a* and *OscpSRP54b* Interact with cpSRP43 In Vivo

**Fig. 9:**
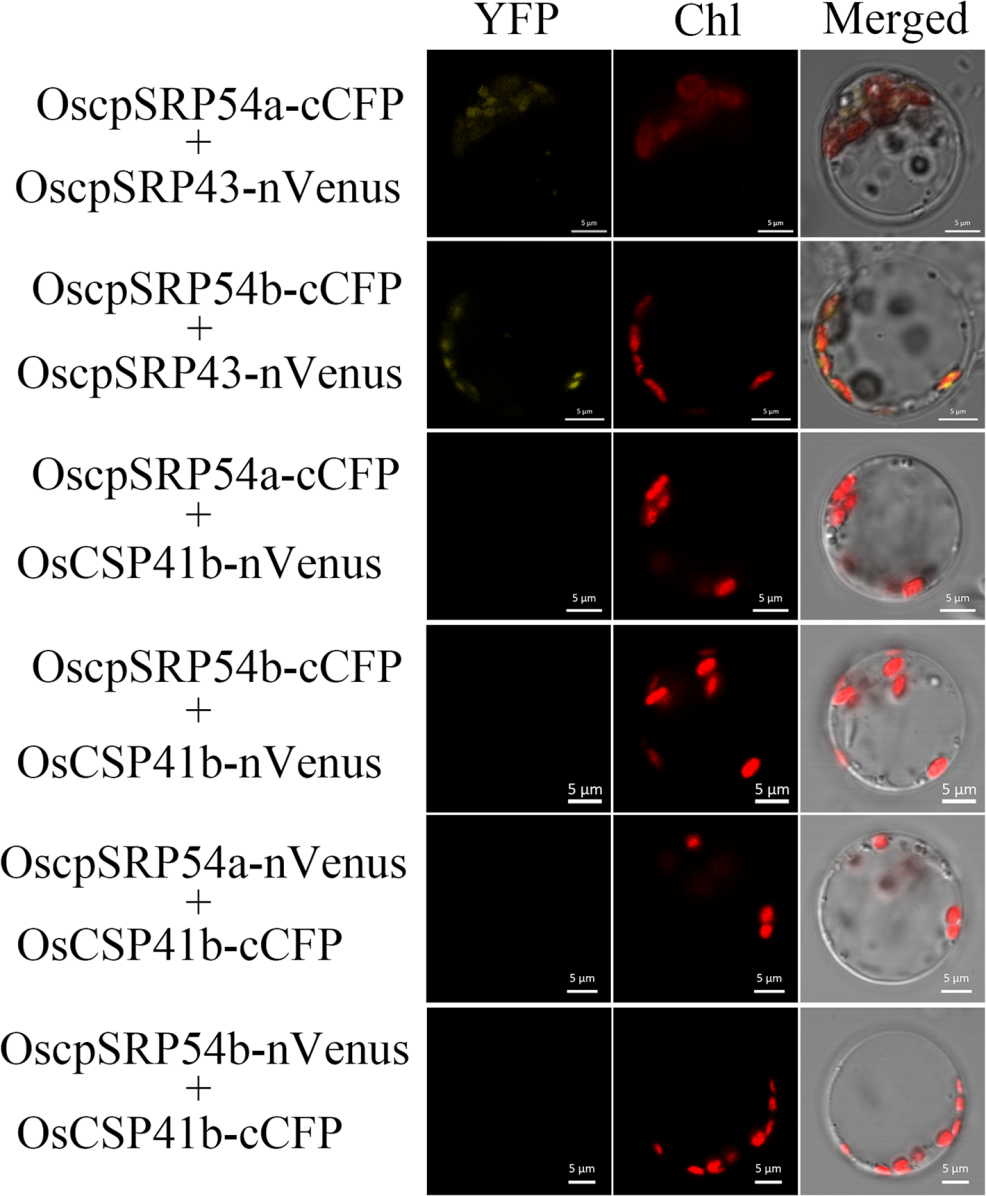
BiFC assay for interaction between OscpSRP54a, OscpSRP54b and OscpSRP43 in rice protoplasts. Green fluorescence indicates GFP signal, red fluorescence indicates chloroplast autofluorescence, and yellow fluorescence indicates the merged signal of green and red fluorescences

It has been shown that AtcpSRP54 interacts with AtcpSRP43 to form the heterodimer which binds to the L18 sequence of LHCPs to form the cpSRP-LHCPs complex for transporting LHCPs to thylakoids (Groves et al. 2001; Goforth et al. 2004). To verify whether OscpSRP54a and OscpSRP54b interact with OscpSRP43, the full length CDS of OscpSRP54a and OscpSRP54b were fused to C-terminal CFP respectively, and the full length CDS of OscpSRP43 was fused to N-terminal Venus. Co-expression of the OscpSRP54a-cCFP and OscpSRP43-nVenus fusion proteins in rice green tissue protoplasts produced obvious YFP signals overlapped with the auto fluorescence of chloroplasts (Fig. 9). The similar result was obtained by co-expression of OscpSRP54b-cCFP and OscpSRP43-nVenus fusion proteins. In contrast, co-expression of OscpSRP54a-cCFP and OsCSP41b-nVenus, or OscpSRP54b-cCFP and OsCSP41b-nVenus did not show the BiFC fluorescence (Fig. 9). Similarly, co-expression of OscpSRP54a-nVenus and OsCSP41b-cCFP, or OscpSRP54b-nVenus and OsCSP41b-cCFP did not show the BiFC fluorescence (Fig. 9). These results clearly demonstrated that both OscpSRP54a and OscpSRP54b interacted with OscpSRP43, respectively.

## Discussion

Deficient chlorophyll contents shown by *Arabidopsis cpSRP* mutants indicate that cpSRP subunits play important roles for chloroplast development (Pilgrim et al. 1998; Amin et al. 1999; Hutin et al. 2002; Walter et al. 2015). Further studies on cpSRP43 and cpSRP54 defective mutants in *Chlamydomonas* and *Arabidopsis* suggest that the cpSRP43/SRP54 complex are able to recognize and bind to the hydrophobic LHCPs during passing through the stroma, thus, the dysfunctional cpSRP43 and/or cpSRP54 disrupt their assembly with LHCPs in thylakoids, leading to impaired chloroplast development (Amin et al. 1999; Hutin et al. 2002; Jeong et al. 2017). In the present study, we identified a chlorophyll-deficient mutant *pgl14*, which possessed a single nucleotide substitution at the splicing site of *OscpSRP54a*, leading to altered splicing transcripts and terminated prematurely. Complementation by the wild type allele could restore the *pgl14* phenotype. OscpSRP54a is a homologue of Arabidopsis cpSRP54 with 70% identity at the amino acid level. The *ffc* mutants defective in AtcpSRP54 show severely yellow true leaves that subsequently become green (Amin et al. 1999). Unlike the *ffc* mutants, *pgl14* exhibited pale green leaf phenotype in the whole life period. It is noticed that the rice mutant *ygl138* has an 18 bp deletion in *OscpSRP54* and shows a similar phenotype to *pgl14*, suggesting that *ygl138(t)* is allelic to *PGL14* (Zhang et al. 2013). Unlike Arabidopsis which possesses only one copy of *cpSRP54*, the rice genome possesses two copies of *cpSRP54s*, *OscpSRP54a* and *OscpSRP54b*, which are physically adjacent and share 78% identity at the protein level. Independent knockout plants of *OscpSRP54b* were seedling lethal but showed pale green leaf phenotype and severely decreased chlorophyll content, resembling to *pgl14*. These results suggest that both *OscpSRP54a* and *OscpSRP54b* are indirectly associated with chlorophyll metabolism probably resulting from the impaired chloroplast development in the mutants.

It has been shown that AtcpSRP54 proteins are equally distributed between thylakoids and stroma by immunolocalization (Hutin et al. 2002). In the present study, we demonstrated that both OscpSRP54a and OscpSRP54b localized to chloroplasts, similar to OscpSRP43 (Lv et al. 2015). The thylakoid membranes in *pgl14* seem to be disrupted, and the grana stacks were thinner than those of the wild-type at the tillering stage (Fig. 3e-g). Nevertheless, *pgl14* is viable and capable of seeding at maturity (Shi et al. 2013). However, more severely destroyed stroma and a large number of hollow vesicles were found in the chloroplasts of the *OscpSRP54b-*knockout lines. The severe and irreversible chloroplast destruction could be the reason for the lethality of *OscpSRP54b-*knockout mutants. These results implicate that functional OscpSRP54b, but not OscpSRP54a, is necessary for rice survival.

Biochemical and genetic studies have indicated that cpSRP54 and cpSRP43 are able to form the heterodimeric complex to participate in localizing LHCPs to thylakoid membranes post-translationally (Hutin et al. 2002; Goforth et al. 2004; Dünschede et al. 2015). Our results suggested that both OscpSRP54a and OscpSRP54b could interact with OscpSRP43, respectively; indicating that OscpSRP54a and OscpSRP54b both functioned similarly as AtcpSRP54 for cpSRP-mediated protein transportation (Amin et al. 1999; Yu et al. 2012). Nevertheless, the existence of an alternative pathway has been confirmed in Arabidopsis that the targeting of LHCPs to thylakoids can be done when cpSRP54 is absent and unable to form the cpSRP-LHCP transit complex (Tzvetkova-Chevolleau et al. 2007). It has been shown that Glutamyl-tRNA reductase (GluTR) is the initial and rate-limiting enzyme for 5-aminolevolinic acid synthesis. Recent studies have demonstrated that cpSRP43 directly binds to GluTR and prevents aggregation of GluTR, thereby enhancing the stability of active GluTR (Wang et al. 2018). In *pgl14* and cr1–2, the transcription levels of *OscpSRP43* were significantly lower than those of the wild types, we speculated that downregulation of cpSRP43 in the mutants could lead to the decrease of stability and catalytic activity of GluTR, thus inhibiting the chlorophyll biosynthesis and ultimately leading to a decreased level of chlorophyll in the mutants. Interestingly, *OscpSRP54b* was significantly upregulated in *pgl14*, whereas *OscpSRP54a* was notably downregulated in cr1–2 mutant (Fig. 8). We speculate that *OscpSRP54b* might partially compensate for the defect of *OscpSRP54a* in *pgl14*, however, the severe destruction of chloroplasts in cr1–2 inhibited the transportation of LHCPs to thylakoids, resulting in the significantly down-regulation of both *OscpSRP54a* and *OscpSRP54b*.

It has been shown that the downregulation of genes involving in chlorophyll biosynthesis, photosynthesis and chloroplast development could be an indirect response to chlorophyll-deficient mutants (Yu et al. 2012; Lv et al. 2015; Qiu et al. 2018). For example, *AtcpSRP54* mutations lead to decreased expression of *AtGLK1*, *AtGLK2* and *GUN4* which are related to plastid-to nucleus retrograde signaling (Lopez-Juez and Pyke 2005). In our study, the expression level of *CHLH* was significantly decreased in *pgl14* and cr1–2. CHLH, a Mg-chelatase H subunit, is a multi-functional protein involved in plastid-to nucleus retrograde signaling and chlorophyll synthesis (Mochizuki et al. 2001; Jung et al. 2003; Wu et al. 2009; Tsuzuki et al. 2011). At*CpSRP54* is found to be associated with chloroplast ribosomes in the stroma, interacts with chloroplast synthesized thylakoid membrane proteins D1 and cytochrome b_6_ to perform its conserved role in co-translational targeting (Nilsson et al. 1999; Nilsson and vanWijk 2002; Piskozub et al. 2015). Similarly, *PsbA* encoding chloroplast D1 protein is significantly downregulated in *pgl14* compared with the wild type. In contrast, the knockout mutant of *OscpSRP54b* induced a notable upregulation in *PsbA* transcription level. Studies in *Synechocystis sp* PCC 6803 have demonstrated that chlorophyll synthase/HliD complex binding with the Ycf39 protein and YidC/Alb3 insertase is involved in the photosystem II assembly, suggesting a link between chlorophyll biosynthesis and the Sec/YidC-dependent cotranslational insertion of nascent photosystem polypeptides into membranes (Chidgey et al. 2014; Knoppová et al. 2014). In our study, the expression level of *YGL1*, the homologue of *Synechocystis sp* PCC 6803 chlorophyll synthase in rice, was significantly downregulated in *pgl14*, but was significantly upregulated in cr1–2, these results implied that OscpSRP54b and OscpSRP54a might play distinct roles in transporting different chloroplast proteins into thylakoids through cpSRP-mediated pathway although the mechanism requires to be furthered studied.

## Methods

### Plant Materials

The *pale green leaf 14* (*pgl14*) mutant was obtained from ethane methyl sulfonate (EMS) mutagenesis of the wild-type (WT) cultivar IR64 (Wu et al. 2005). The pale green phenotype is controlled by a single recessive nuclear gene (Shi et al. 2013). Normal green leaf cultivar Moroberekan was used as the male parent to cross with *pgl14* for construction of an F_2_ fine mapping population. The parents and the population were grown under natural summer conditions in the paddy field at the China National Rice Research Institute (CNRRI), Hangzhou, China. The transgenic plants and WT were grown in the greenhouse at 30 °C and humidity of ca.70 with 16 h light/8 h dark cycle at CNRRI.

### Measurement of Pigment Content

The total chlorophylls (Chl) were extracted from 10 mg fresh leaves with 95% alcohol in darkness for 48 h. The extracts were measured spectrophotometrically at 470 nm, 645 nm and 663 nm with a SpectraMax i3x Multi-Mode Microplate Reader (Molecular Devices, USA). Total Chl contents were determined according to the method of Arnon (Arnon 1949), and total carotenoid contents were determined as described by Wellburn (Wellburn 1994). All experiments were carried out with three biological replicates. Student’s *t*-test was conducted using EXCEL2013 and Duncan’s test was conducted by SAS 9.0. Means from three replicates were used for analysis.

### Transmission Electron Microscopy Analysis

Full expanded leaves of *pgl14*, WT and C-*pgl14* were collected at the seedling stage, while full expanded leaves of Kitaake, *cpSRP54b* knockout plants cr1–2 and cr2–3 were collected 1 week after transplanting. Leaf sections were fixed with 2.5% glutaraldehyde in phosphate buffer (pH 7.2) for 16 h at 4 °C, followed by rinsing, dewatering, embedding, and staining according to the method described by Lv et al. (2015). The chloroplast ultrastructure was observed by a Tecnai G^2^ F20 S-TWIN transmission electron microscope at Zhejiang University, Hangzhou, China.

### Map-Based Cloning of *PGL14* and Complementation Assay

The mutation was previously mapped to a 299 kb region in chromosome 11 (Shi et al. 2013). A total of 1008 mutant type F_2_ individuals derived from the cross *pgl14*/Moroberekan were used for fine mapping using simple sequence repeat (SSR) markers (Supplementary Table S1).

The genomic DNA was extracted following the minipreparation method (Lu and Zheng 1992). The genomic DNA fragments of the candidate gene were amplified from WT and *pgl14*, then sequenced and compared using DNASTAR software. RT-PCR analysis was used to confirm the splicing site in WT and *pgl14* using the primers PTs (Supplementary Table S2). The sequences of the genomic DNA fragments and the transcripts were determined at Shanghai Invitrogen Inc. (Shanghai, China).

For functional complementation, a 9.6 kb WT genomic fragment containing a 4.5 kb entire open reading frame (ORF) of *PGL14*, a 3.6 kb upstream region, and a 1.5 kb downstream region was amplified using the specific primers PQf (Supplementary Table S2). The PCR products were double-digested with *BamH* I and *Kpn* I, and the fragments were recovered using the Axygen DNA gel extraction kit (Axygen scientific, USA). Then, the fragments were cloned into the binary vector pCAMBIA1300 to form a new transformation construct, cPGL. The new construct was introduced into the embryogenic calli generated from the mature seed embryos of *pgl14* using *Agrobacterium-*mediated transformation method (Hiei and Komari 2008).

### CRISPR/Cas9-Mediated Editing

To generate *OscpSRP54b* knockout mutants, two target sequences (TGGGCAGCTCACGACCGGGC, CCAACAGTTATCCTATTGG) were designed using CRISPR-P (Lei et al. 2014). The guide sequences were inserted into commercial gRNA expression vector VK005–01 respectively to create two new CRISPR/Cas9 vectors cr1 and cr2 (Supplementary Fig. 3) following the manufacture’s instruction (ViewSolid Biotech, Beijing). The cr1 and cr2 constructs were respectively introduced into Kitaake mature embryo-induced calli by *Agrobacterium-*mediated transformation (Hiei and Komari 2008).

### Sequence Alignment and Phylogenetic Analysis

BlastP (https://www.ncbi.nlm.nih.gov/) was used to search homologous protein sequences of OscpSRP54a. The homologous sequences were aligned using the BioEdit software. The neighbor-joining phylogenetic tree was constructed using MEGA 5.1. 1000 bootstrap replicates were used for statistical support for the node values.

### Quantitative Reverse Transcription PCR

Total RNA was extracted using the TRIzol method following the manufacture’s instruction (Invitrogen, USA). For RNA isolation, the roots and shoots of WT and Kitaake were collected from 10 day-old seedlings, the top full expanded leaves, leaf sheaths, roots, root stems, nodes and internodes of WT and Kitaake were collected from 10 week-old plants, roots, basals, nodes, internodes, flag leaves, flag leaf sheaths and panicles of WT and Kitaake were collected at the grain filling stage. For quantitative reverse transcription (qRT-PCR) analysis of genes associated with chlorophyll biosynthesis and chloroplast development, total RNA was extracted from the top full expanded leaves of *pgl14* and WT at 3 weeks after sowing. Total RNA was extracted from 1 week-old leaves of cr1–2 and Kitaake. The first-strand cDNA was synthesized using the First Strand cDNA synthesis kit following the manufacturer’s protocol (TOYOBO Biotech, Japan). qRT-PCR was performed in a total volume of 20 μL qRT-PCR reaction buffer containing 2 μL reverse-transcribed product, 0.2 μM of each primer, and 2 × PowerUp SYBR Green PCR Master Mix (ThermoFisher Scientific, USA), on a Thermal Cycle Dice TM Real Time System II (Takara Biotech, Japan) with a cycling program of 2 m at 50 °C, 2 m at 95 °C, followed by 40 cycles of 15 s at 95 °C, 15 s at 55 °C, and 60 s at 72 °C. The *ubiquitin* gene (*LOC_Os03g13170*, *Ubq*) was used as an internal control. Primers used for qRT-PCR are listed in Supplementary Table S3. The means from three biological replicates were used for analysis by Student’s *t*-test and Duncan’s test by EXCEL2013 and SAS 9.0, respectively. The 2^–ΔΔCT^ method was used to determine the relative transcript levels in gene expression.

### Subcellular Localization and Bimolecular Fluorescence Complementation Assay

To determine the subcellular localization of OscpSRP54a and OscpSRP54b, their full length CDSs were amplified using the specific primers SLPGL14 and SLCr1, respectively (Supplement Table S2). The PCR products were double-digested with *Xba* I and *Bam*H I, and the fragments were inserted into the 5′-terminal of GFP driven by the CaMV 35S promoter in the transient expression vector PAN580 to form the new constructs, PAN580-OscpSRP54a and PAN580-OscpSRP54b, respectively. For BiFC assay, the full length CDSs of OscpSRP54a and OscpSRP54b were amplified using the specific primers BiPGL14, and BiCr1. The full length CDS of *OsCSP41b*, which encodes for a chloroplast-localized protein (Mei et al. 2017), was amplified using the primer BiCSP41b and fused with cCFP and nVenus fragment as a control. The PCR products were double-digested with *Kpn* I and *Bam*H I, and the fragments were inserted to the 5′-terminal of cCFP driven by the CaMV 35S promoter in the expression vector pE3449 to form three new constructs, OscpSRP54a-cCFP, OscpSRP54b-cCFP and OsCSP41b-cCFP, respectively. These fragments were inserted to the 5′-terminal of nVenus in pE3308 to form OscpSRP54a-nVenus, OscpSRP54b-nVenus and OsCSP41b-nVenus constructs. The full length CDS of *OscpSRP43* was amplified using the primers BiW67 (Supplemental Table S2), and double-digested with *Kpn* I and *Sma* I, then the fragments were inserted to the 5′-terminal of nVenus driven by the 35S promoter in pE3308 to generate a new construct OscpSRP43-nVenus. The constructs were transformed into rice protoplasts according to the protocol described previously (Zhang et al. 2011).

## Conclusions

*OscpSRP54a* and *OscpSRP54b* encode two homologous chloroplast signal recognition particles and their loss of function led to pale green leaves. Both OscpSRP54a and OscpSRP54b localize to the chloroplast and are able to interact with OscpSRP43, respectively. These results will facilitate efforts to further uncover the molecular mechanism of chloroplast protein transporting in monocots.

## Supplementary information

**Additional file 1 Supplementary Fig. 1** Subcellular localization prediction of OscpSRP54a and OscpSRP54b using ChloroP program. **Supplementary Fig. 2** Expression of genes associated with chlorophyll biosynthesis and chloroplast development. **Supplementary Fig. 3** Schematic structure of cr1 and cr2. **Supplementary Table S1** Details of SSR markers for fine mapping of *pgl14*. **Supplementary Table S2** Primer sequences for vector construction and reverse transcription-PCR. **Supplementary Table S3** Primer sequences for RT-PCR.

## Data Availability

Not applicable.

## Abbreviations

Chl: Chlorophyll
cpSRP: Chloroplast signal recognition particle
cpFtsY: Chloroplast signal recognition particle receptor
TLA: Truncated light-harvesting chlorophyll antenna
LHCPs: Light harvesting chlorophyll a/b binding proteins
CRISPR/Cas9: Clustered regularly interspaced short palindromic repeats and CRISPR-associated protein 9
