# Supplementary material for: Chloroplast SRP54s are Essential for Chloroplast Development in Rice
Source: Rice (N Y). 2020 Aug 6;13:54. doi: 10.1186/s12284-020-00415-2 (PMC7410889; doi:10.1186/s12284-020-00415-2)
Supplement: Supplementary file 1 — Additional file 1 Supplementary Fig. 1 Subcellular localization prediction of OscpSRP54a and OscpSRP54b using ChloroP program. Supplementary Fig. 2 Expression of genes associated with chlorophyll biosynthesis and chloroplast development. Supplementary Fig. 3 Schematic structure of cr1 and cr2. Supplementary Table S1 Details of SSR markers for fine mapping of pgl14. Supplementary Table S2 Primer sequences for vector construction and reverse transcription-PCR. Supplementary Table S3 Primer sequences for RT-PCR. [file 12284_2020_415_MOESM1_ESM.pdf]

ChloroP 1.1 Server - prediction results

Technical University of Denmark

```
### chlorop v1.1 prediction results ##########################
Number of query sequences: 2

Name                    Length       Score  cTP         CS-    cTP-
                                                      score  length
-----------------------------------------------------------------
OscpSRP54a                 550       0.590   Y      11.938     68
OscpSRP54b                 557       0.559   Y      11.938     73
-----------------------------------------------------------------
```

Supplementary Fig. 1 Subcellular localization prediction of OscpSRP54a and OscpSRP54b using ChloroP program. Chloroplast transit peptides (cTP) were identified in the sequences of OscpSRP54a and OscpSRP54b.

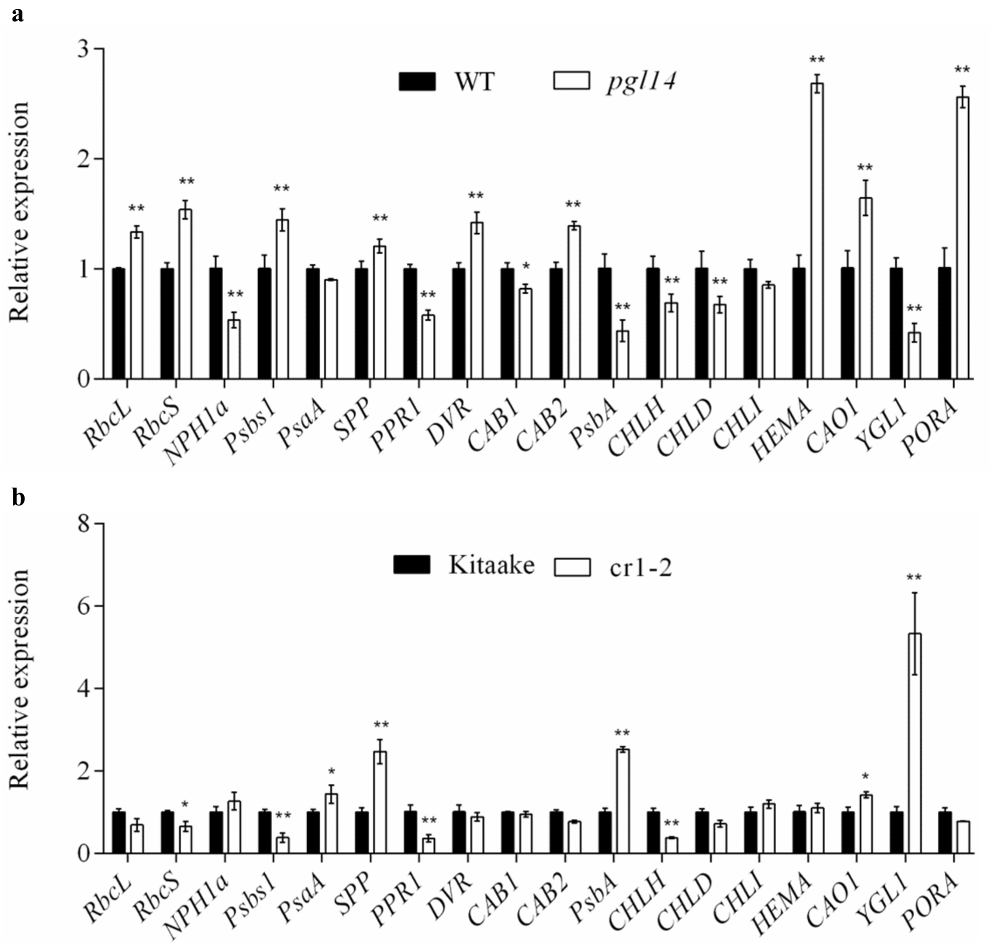

Supplementary Fig. 2 Expression of genes associated with chlorophyll biosynthesis and chloroplast development.

(**a**) The expression of genes associated with chlorophyll biosynthesis and chloroplast development in *pgl14* and WT. (**b**) The expression of genes associated with chlorophyll biosynthesis and chloroplast development in cr1-2 and wild type Kitaake. The transcript levels of all tested genes are normalized to the levels of *Ubq* (*LOC_Os03g13170*). The values are means ± SD (n = 3). * indicates significance at $p \leq 0.05$; ** indicates significance at $p \leq 0.01$ by Student's t-test.

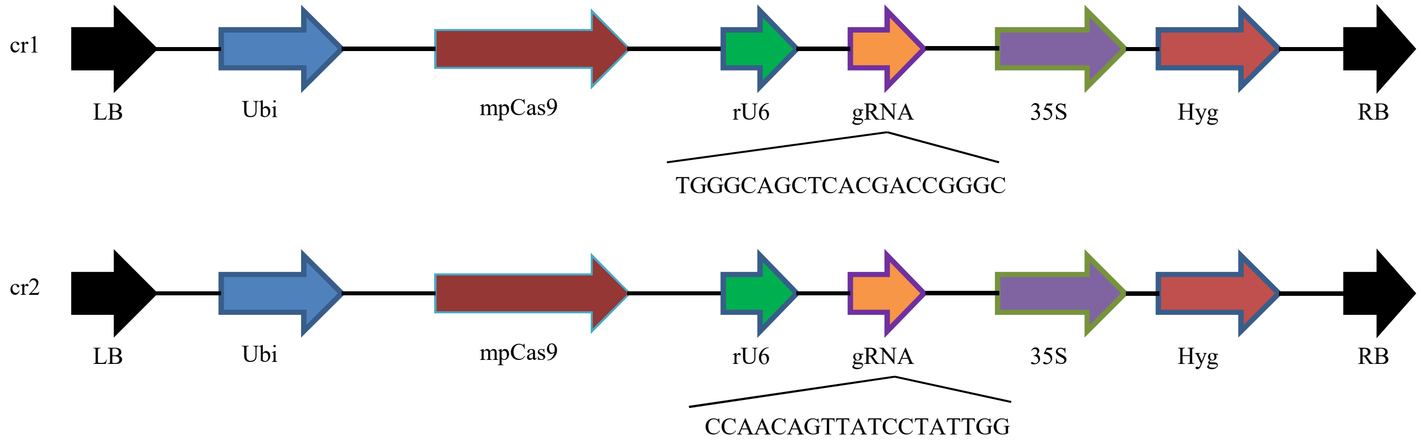

Supplementary Fig. 3 Schematic structure of cr1 and cr2.
LB, Vector left border; Ubi, Ubi promoter; mpCas9, Cas9 protein; rU6, Rice U6 promoter; gRNA, Guide RNA; 35S, 35S promoter; Hyg, Hygromycin; RB, Vector right border.

Supplementary Table S1 details of SSR markers for fine mapping of *pgl14*

Markers	sequence
RM1812F	CCTACCTCCAGTGAGAGCTAACC
RM1812R	ACGTGCATTTGTGTGGTTTAGG
RM26092F	GTCATCGTCGTGGTCGCAGTCC
RM26092R	TCTGGAGTGGCGTTGACTGTTGC
RM26076F	CCTTCTCTCTCCCACATCTCTAGC
RM26076R	CTAGTGAGTCCCACGTGTCAACC
RM26079F	CTTGACGACCTGACCCAAATACC
RM26079R	GTCAATCGTTAGCCATGTGTTGG
RM26085F	GAGTTCCAACCGTGTGTTCTTCC
RM26085R	CCGCTATTTACTCCGTCAGTTGG

Supplementary Table S2 Primer sequences for vector construction and reverse transcription-PCR

Primer	sequence
PTsF	TCGCTCATCGGCAATGGA
PTsR	TTCCACGGCATTCTTGGTTATT
PQfF	CGCGGATCCTGGCTCCCACTTGTCAAATAGAT
PQfR	CGGGGTACCAATACTACCCTGGAAAGAGTGTTGTC
SLPGL14F	TCTAGAATGGAGGCCACGGCGCTCAC
SLPGL14R	GGATCCAGAACTAGGGACTGCATCCAGC
SLCr1F	TCTAGAATGGAGGCCACCAGTAGTAC
SLCr1R	GGATCCTCGTCGACGGAAACCGCGCA
BiPGL14F	GGGGTACCTCATGGAGGCCACGGCGCTCAC
BiPGL14R	CGGGATCCCAGAACTAGGGACTGCATCCAGC
BiCr1F	GGGGTACCTCATGGAGGCCACCAGTAGTAC
BiCr1R	CGGGATCCCTCGTCGACGGAAACCGCGCA
BiCSP41bF	GGGGTACCTCATGGCAGCAACAGCCTCCCT
BiCSP41bR	CGGGATCCCGACGCTGACGAGCTTCTT
BiW67F	GGGGTACCTCATGGAGGCTGTCCTACGACA
BiW67R	TCCCCCGGGCCCGGCGACCGGCGGCGG

Supplementary Table S3 Primer sequences for qRT-PCR

Marker	Forward sequence	Reverse sequence
OscpSRP43	AGAGAACTCCAGCCCTCTCC	GGACACAATACGGTCCACCT
OscpSRP54a	AAAGAGTTGTATGCTGGTCG	TTTGGCTTCTGTTCTTTCT
OscpSRP54b	GATGGAGGAGGAAGAGGGGA	GCGATGTTGTCCTTCGTCAG
RbcL	CTTGGCAGCATTCCGAGTAA	ACAACGGGCTCGATGTGATA
RbcS	TCCGCTGAGTTTTGGCTATTT	GGACTTGAGCCCTGGAAGG
PsaA	GCGAGCAAATAAAACACCTTTC	GTACCAGCTTAACGTGGGGAG
SPP	CGGAGAGGAAACATAATGAC	ATAGGCATTTGTCTTTGTCTC
NPH1a	CACTTGCAACCAATGCGTGA	ATCCGGGAGTTCCTTTGCAG
PPR1	CTAAGACCGAATGACAAATGC	GCACTGCCAACAAGAATACC
Psbs1	CTGAGCCGAAGCCAAAGTTC	ATCCCCGTCTCCAGGTTCAG
DVR	CGAGCCCAGGTTCATCAAGGTGC	CCTCCCGATCTTGCCGAACTCC
Cab1	AGATGGGTTTAGTGCGACGAG	TTTGGGATCGAGGGAGTATTT
Cab2	TGTTCTCCATGTTCGGCTTCT	GCTACGGTCCCCACTTCACT
psbA	CCCTCATTAGCAGATTCGTTTT	ATGATTGTATTCCAGGCAGAGC
CHLI	AGTAACCTTGGTGCTGTG	AATCCATCAACATTCAACTCTG
CHLD	GGAAAGAGAGGGCATTAG	CAATACGATCAAGTAAGTGTT
CHLH	CTATACATTCGCCACACT	TATCACACAACTCCCAAG
HEMA1	CGCTATTTCTGATGCTATGGGT	TCTTGGGTGATGATTGTTTGG
CAO1	GATCCATACCCGATCGACAT	CGAGAGACATCCGGTAGAGC
YGL1	CAGTCTCCAATGGCCACCT	TGCTTTCATCAGTGGCTGG
PORA	TGTACTGGAGCTGGAACAACAA	GAGCACAGCAAAATCCTAGACG
Ubq	CCCTCCACCTCGTCCTCAG	AGATAACAACGGAAGCATAAAAGTC
